# Supplementary material for: Modeling glioblastoma heterogeneity as a dynamic network of cell states
Source: Mol Syst Biol. 2021 Sep 16;17(9):e10105. doi: 10.15252/msb.202010105 (PMC8444284; doi:10.15252/msb.202010105)
Supplement: Supplementary file 6 — Source Data for Figure 5 [file MSB-17-e10105-s004.zip › Figure5A_sourcedata/GSEA_3017/nosigdb_stateE.GseaPreranked.1622795031093/RICHARDS_NOWAKOWSKI_SCIENCE_2017_IPC_DIV1_UPREG.html]

Details for gene set RICHARDS\_NOWAKOWSKI\_SCIENCE\_2017\_IPC\_DIV1\_UPREG[GSEA]

|  || Dataset | state63017 |
| Phenotype | NoPhenotypeAvailable |
| Upregulated in class | na\_neg |
| GeneSet | RICHARDS\_NOWAKOWSKI\_SCIENCE\_2017\_IPC\_DIV1\_UPREG |
| Enrichment Score (ES) | -0.6115008 |
| Normalized Enrichment Score (NES) | -3.6437793 |
| Nominal p-value | 0.0 |
| FDR q-value | 0.0 |
| FWER p-Value | 0.0 |
Table: GSEA Results Summary

  

Fig 1: Enrichment plot: RICHARDS\_NOWAKOWSKI\_SCIENCE\_2017\_IPC\_DIV1\_UPREG      
 Profile of the Running ES Score & Positions of GeneSet Members on the Rank Ordered List

  

| PROBE | GENE SYMBOL | GENE\_TITLE | RANK IN GENE LIST | RANK METRIC SCORE | RUNNING ES | CORE ENRICHMENT || 1 | TAGLN2 |  |  | 32 | 0.621 | -0.0483 | No |
| 2 | FRMD4B |  |  | 48 | 0.532 | -0.0696 | No |
| 3 | ENC1 |  |  | 58 | 0.501 | -0.0814 | No |
| 4 | TMSB4X |  |  | 93 | 0.412 | -0.1341 | No |
| 5 | EFNB2 |  |  | 94 | 0.411 | -0.1318 | No |
| 6 | EZR |  |  | 96 | 0.410 | -0.1311 | No |
| 7 | STK17A |  |  | 97 | 0.408 | -0.1288 | No |
| 8 | EML1 |  |  | 110 | 0.383 | -0.1461 | No |
| 9 | LGALS1 |  |  | 113 | 0.368 | -0.1473 | No |
| 10 | DAAM1 |  |  | 138 | 0.343 | -0.1842 | No |
| 11 | NUDT4 |  |  | 146 | 0.337 | -0.1936 | No |
| 12 | NFIX |  |  | 171 | 0.317 | -0.2307 | No |
| 13 | CUX1 |  |  | 188 | 0.306 | -0.2549 | No |
| 14 | CALM2 |  |  | 191 | 0.303 | -0.2564 | No |
| 15 | CSRP2 |  |  | 247 | 0.264 | -0.3439 | No |
| 16 | HMGCR |  |  | 251 | 0.261 | -0.3473 | No |
| 17 | FDFT1 |  |  | 266 | 0.252 | -0.3686 | No |
| 18 | TNPO2 |  |  | 273 | -0.251 | -0.3769 | No |
| 19 | ARHGAP11B |  |  | 277 | -0.252 | -0.3803 | No |
| 20 | TUBA1C |  |  | 282 | -0.253 | -0.3854 | No |
| 21 | ZMYM1 |  |  | 283 | -0.253 | -0.3840 | No |
| 22 | NUP37 |  |  | 286 | -0.255 | -0.3858 | No |
| 23 | ZNF850 |  |  | 291 | -0.257 | -0.3908 | No |
| 24 | H2AFY |  |  | 297 | -0.257 | -0.3975 | No |
| 25 | RAN |  |  | 298 | -0.257 | -0.3960 | No |
| 26 | CCDC77 |  |  | 306 | -0.259 | -0.4059 | No |
| 27 | ZNF682 |  |  | 309 | -0.260 | -0.4077 | No |
| 28 | TGIF1 |  |  | 318 | -0.263 | -0.4192 | No |
| 29 | PSIP1 |  |  | 319 | -0.263 | -0.4177 | No |
| 30 | TUBG1 |  |  | 323 | -0.263 | -0.4211 | No |
| 31 | MAGOHB |  |  | 328 | -0.264 | -0.4261 | No |
| 32 | NUDCD2 |  |  | 330 | -0.264 | -0.4262 | No |
| 33 | RCC2 |  |  | 340 | -0.266 | -0.4393 | No |
| 34 | BRD8 |  |  | 341 | -0.266 | -0.4378 | No |
| 35 | ARHGEF39 |  |  | 346 | -0.268 | -0.4428 | No |
| 36 | KNTC1 |  |  | 352 | -0.269 | -0.4494 | No |
| 37 | POLR2A |  |  | 361 | -0.272 | -0.4608 | No |
| 38 | KIAA0586 |  |  | 362 | -0.272 | -0.4593 | No |
| 39 | DLEU2 |  |  | 365 | -0.272 | -0.4610 | No |
| 40 | APOLD1 |  |  | 374 | -0.275 | -0.4724 | No |
| 41 | TBC1D31 |  |  | 380 | -0.276 | -0.4790 | No |
| 42 | IVNS1ABP |  |  | 381 | -0.276 | -0.4774 | No |
| 43 | TMEM237 |  |  | 383 | -0.276 | -0.4775 | No |
| 44 | HNRNPA2B1 |  |  | 391 | -0.279 | -0.4873 | No |
| 45 | CCAR1 |  |  | 396 | -0.282 | -0.4922 | No |
| 46 | DBF4B |  |  | 399 | -0.283 | -0.4938 | No |
| 47 | LSM4 |  |  | 406 | -0.284 | -0.5019 | No |
| 48 | MAGI1 |  |  | 413 | -0.287 | -0.5100 | No |
| 49 | ARHGAP19 |  |  | 414 | -0.287 | -0.5084 | No |
| 50 | ZNF273 |  |  | 415 | -0.288 | -0.5068 | No |
| 51 | NIF3L1 |  |  | 418 | -0.289 | -0.5084 | No |
| 52 | SCLT1 |  |  | 419 | -0.289 | -0.5068 | No |
| 53 | MYBL1 |  |  | 432 | -0.294 | -0.5246 | No |
| 54 | CTCF |  |  | 443 | -0.297 | -0.5391 | No |
| 55 | ZNF85 |  |  | 444 | -0.298 | -0.5375 | No |
| 56 | PLCB1 |  |  | 446 | -0.298 | -0.5374 | No |
| 57 | ZNF43 |  |  | 449 | -0.299 | -0.5390 | No |
| 58 | CEP57L1 |  |  | 450 | -0.299 | -0.5373 | No |
| 59 | TRIM28 |  |  | 460 | -0.304 | -0.5502 | No |
| 60 | PNRC2 |  |  | 461 | -0.304 | -0.5485 | No |
| 61 | CNTRL |  |  | 462 | -0.304 | -0.5468 | No |
| 62 | CCNG2 |  |  | 467 | -0.305 | -0.5515 | No |
| 63 | SUV39H2 |  |  | 469 | -0.305 | -0.5515 | No |
| 64 | NEIL3 |  |  | 475 | -0.307 | -0.5578 | No |
| 65 | NUMA1 |  |  | 477 | -0.307 | -0.5577 | No |
| 66 | EXOSC9 |  |  | 481 | -0.308 | -0.5609 | No |
| 67 | ATCAY |  |  | 486 | -0.309 | -0.5656 | No |
| 68 | RSRC1 |  |  | 494 | -0.312 | -0.5752 | No |
| 69 | ILF2 |  |  | 497 | -0.313 | -0.5767 | No |
| 70 | CEP97 |  |  | 499 | -0.313 | -0.5765 | No |
| 71 | PA2G4 |  |  | 501 | -0.314 | -0.5764 | No |
| 72 | COX17 |  |  | 505 | -0.316 | -0.5795 | No |
| 73 | HNRNPH3 |  |  | 507 | -0.316 | -0.5793 | No |
| 74 | PIK3R3 |  |  | 508 | -0.317 | -0.5776 | No |
| 75 | SRRT |  |  | 509 | -0.317 | -0.5758 | No |
| 76 | BARD1 |  |  | 510 | -0.318 | -0.5740 | No |
| 77 | DNA2 |  |  | 513 | -0.318 | -0.5755 | No |
| 78 | CCDC150 |  |  | 516 | -0.319 | -0.5769 | No |
| 79 | SAPCD2 |  |  | 517 | -0.319 | -0.5752 | No |
| 80 | KIF20A |  |  | 523 | -0.321 | -0.5815 | No |
| 81 | SUZ12 |  |  | 524 | -0.322 | -0.5797 | No |
| 82 | SESN3 |  |  | 526 | -0.322 | -0.5795 | No |
| 83 | RHEB |  |  | 528 | -0.323 | -0.5793 | No |
| 84 | CEP192 |  |  | 531 | -0.324 | -0.5807 | No |
| 85 | CEP135 |  |  | 532 | -0.325 | -0.5789 | No |
| 86 | ACTL6A |  |  | 533 | -0.325 | -0.5771 | No |
| 87 | RFC5 |  |  | 534 | -0.326 | -0.5753 | No |
| 88 | TPRKB |  |  | 540 | -0.329 | -0.5815 | No |
| 89 | ING1 |  |  | 541 | -0.329 | -0.5797 | No |
| 90 | H3F3A |  |  | 542 | -0.329 | -0.5778 | No |
| 91 | JADE1 |  |  | 543 | -0.329 | -0.5760 | No |
| 92 | DCLRE1C |  |  | 544 | -0.330 | -0.5741 | No |
| 93 | CDC25C |  |  | 549 | -0.332 | -0.5788 | No |
| 94 | GPSM2 |  |  | 550 | -0.334 | -0.5769 | No |
| 95 | PLK4 |  |  | 551 | -0.334 | -0.5750 | No |
| 96 | HAUS6 |  |  | 555 | -0.337 | -0.5780 | No |
| 97 | C2orf69 |  |  | 556 | -0.338 | -0.5761 | No |
| 98 | SAP30 |  |  | 558 | -0.339 | -0.5758 | No |
| 99 | FAM83D |  |  | 559 | -0.340 | -0.5739 | No |
| 100 | TROAP |  |  | 562 | -0.341 | -0.5753 | No |
| 101 | CEP128 |  |  | 565 | -0.342 | -0.5766 | No |
| 102 | SASS6 |  |  | 567 | -0.342 | -0.5763 | No |
| 103 | LSM5 |  |  | 568 | -0.342 | -0.5744 | No |
| 104 | HMGB3 |  |  | 577 | -0.348 | -0.5854 | No |
| 105 | RCC1 |  |  | 582 | -0.350 | -0.5899 | No |
| 106 | GEN1 |  |  | 584 | -0.351 | -0.5896 | No |
| 107 | NUP107 |  |  | 586 | -0.352 | -0.5892 | No |
| 108 | DDX39A |  |  | 590 | -0.353 | -0.5921 | No |
| 109 | CDK5RAP2 |  |  | 591 | -0.353 | -0.5901 | No |
| 110 | CENPC |  |  | 596 | -0.356 | -0.5946 | No |
| 111 | HIRIP3 |  |  | 603 | -0.359 | -0.6023 | No |
| 112 | FANCB |  |  | 606 | -0.361 | -0.6035 | No |
| 113 | PRIM2 |  |  | 607 | -0.362 | -0.6015 | No |
| 114 | PARP2 |  |  | 609 | -0.362 | -0.6011 | No |
| 115 | POC1A |  |  | 611 | -0.363 | -0.6007 | No |
| 116 | KIF18B |  |  | 612 | -0.363 | -0.5987 | No |
| 117 | BTG3 |  |  | 615 | -0.365 | -0.5999 | No |
| 118 | RFWD3 |  |  | 617 | -0.365 | -0.5994 | No |
| 119 | C18orf54 |  |  | 619 | -0.366 | -0.5990 | No |
| 120 | ZGRF1 |  |  | 620 | -0.367 | -0.5970 | No |
| 121 | G2E3 |  |  | 624 | -0.371 | -0.5997 | No |
| 122 | WDR76 |  |  | 626 | -0.373 | -0.5993 | No |
| 123 | LCORL |  |  | 627 | -0.374 | -0.5972 | No |
| 124 | NEK2 |  |  | 628 | -0.374 | -0.5951 | No |
| 125 | EXOSC8 |  |  | 630 | -0.376 | -0.5946 | No |
| 126 | RNASEH2A |  |  | 633 | -0.378 | -0.5957 | No |
| 127 | CCP110 |  |  | 636 | -0.381 | -0.5968 | No |
| 128 | SEZ6L |  |  | 642 | -0.383 | -0.6028 | No |
| 129 | PHIP |  |  | 643 | -0.384 | -0.6006 | No |
| 130 | MPHOSPH9 |  |  | 644 | -0.384 | -0.5985 | No |
| 131 | TTF2 |  |  | 646 | -0.386 | -0.5980 | No |
| 132 | GAS2L3 |  |  | 647 | -0.388 | -0.5958 | No |
| 133 | PHF19 |  |  | 648 | -0.391 | -0.5936 | No |
| 134 | ZEB1 |  |  | 653 | -0.396 | -0.5979 | No |
| 135 | SHCBP1 |  |  | 655 | -0.398 | -0.5973 | No |
| 136 | SEPHS1 |  |  | 661 | -0.401 | -0.6031 | No |
| 137 | RHNO1 |  |  | 664 | -0.406 | -0.6041 | No |
| 138 | SAE1 |  |  | 668 | -0.408 | -0.6066 | No |
| 139 | SOGA1 |  |  | 672 | -0.411 | -0.6092 | Yes |
| 140 | KIF18A |  |  | 673 | -0.412 | -0.6069 | Yes |
| 141 | NUDT1 |  |  | 674 | -0.413 | -0.6046 | Yes |
| 142 | GNG4 |  |  | 676 | -0.415 | -0.6039 | Yes |
| 143 | NUP50 |  |  | 677 | -0.415 | -0.6016 | Yes |
| 144 | CIT |  |  | 680 | -0.418 | -0.6025 | Yes |
| 145 | RBMX |  |  | 682 | -0.421 | -0.6017 | Yes |
| 146 | H2AFV |  |  | 684 | -0.423 | -0.6010 | Yes |
| 147 | CCDC14 |  |  | 685 | -0.423 | -0.5986 | Yes |
| 148 | CENPO |  |  | 686 | -0.424 | -0.5963 | Yes |
| 149 | CENPQ |  |  | 689 | -0.425 | -0.5971 | Yes |
| 150 | STIL |  |  | 692 | -0.425 | -0.5980 | Yes |
| 151 | CKAP5 |  |  | 694 | -0.426 | -0.5972 | Yes |
| 152 | SLC25A5 |  |  | 695 | -0.426 | -0.5948 | Yes |
| 153 | DIAPH3 |  |  | 696 | -0.427 | -0.5925 | Yes |
| 154 | RANGAP1 |  |  | 698 | -0.429 | -0.5917 | Yes |
| 155 | ANP32B |  |  | 699 | -0.431 | -0.5893 | Yes |
| 156 | E2F3 |  |  | 700 | -0.431 | -0.5869 | Yes |
| 157 | GADD45G |  |  | 701 | -0.431 | -0.5844 | Yes |
| 158 | CENPL |  |  | 703 | -0.432 | -0.5837 | Yes |
| 159 | CHEK1 |  |  | 704 | -0.433 | -0.5812 | Yes |
| 160 | PARPBP |  |  | 705 | -0.434 | -0.5788 | Yes |
| 161 | TIMELESS |  |  | 707 | -0.435 | -0.5780 | Yes |
| 162 | MZT1 |  |  | 711 | -0.437 | -0.5804 | Yes |
| 163 | ZNF93 |  |  | 712 | -0.438 | -0.5780 | Yes |
| 164 | LMNB2 |  |  | 713 | -0.438 | -0.5755 | Yes |
| 165 | RAD18 |  |  | 715 | -0.443 | -0.5746 | Yes |
| 166 | SKA1 |  |  | 719 | -0.447 | -0.5770 | Yes |
| 167 | MASTL |  |  | 721 | -0.451 | -0.5761 | Yes |
| 168 | TTK |  |  | 724 | -0.454 | -0.5768 | Yes |
| 169 | POLQ |  |  | 726 | -0.455 | -0.5759 | Yes |
| 170 | LINC01224 |  |  | 727 | -0.455 | -0.5733 | Yes |
| 171 | RAD21 |  |  | 728 | -0.458 | -0.5708 | Yes |
| 172 | SPAG5 |  |  | 729 | -0.458 | -0.5682 | Yes |
| 173 | CCNF |  |  | 732 | -0.460 | -0.5689 | Yes |
| 174 | BRIP1 |  |  | 734 | -0.461 | -0.5679 | Yes |
| 175 | ZNF714 |  |  | 735 | -0.461 | -0.5653 | Yes |
| 176 | LBR |  |  | 739 | -0.463 | -0.5676 | Yes |
| 177 | SMC3 |  |  | 743 | -0.470 | -0.5698 | Yes |
| 178 | NUCKS1 |  |  | 744 | -0.471 | -0.5672 | Yes |
| 179 | PTMA |  |  | 745 | -0.475 | -0.5646 | Yes |
| 180 | CHD7 |  |  | 746 | -0.480 | -0.5619 | Yes |
| 181 | TK1 |  |  | 747 | -0.480 | -0.5592 | Yes |
| 182 | MIS18A |  |  | 750 | -0.483 | -0.5597 | Yes |
| 183 | KNSTRN |  |  | 751 | -0.485 | -0.5570 | Yes |
| 184 | PRR11 |  |  | 755 | -0.488 | -0.5591 | Yes |
| 185 | LRRN1 |  |  | 756 | -0.490 | -0.5564 | Yes |
| 186 | SPDL1 |  |  | 758 | -0.491 | -0.5553 | Yes |
| 187 | CDCA3 |  |  | 759 | -0.497 | -0.5525 | Yes |
| 188 | MMS22L |  |  | 761 | -0.500 | -0.5513 | Yes |
| 189 | DCP2 |  |  | 762 | -0.500 | -0.5485 | Yes |
| 190 | GMNN |  |  | 766 | -0.510 | -0.5505 | Yes |
| 191 | RTKN2 |  |  | 767 | -0.511 | -0.5477 | Yes |
| 192 | KIF5B |  |  | 770 | -0.515 | -0.5480 | Yes |
| 193 | CMC2 |  |  | 771 | -0.518 | -0.5451 | Yes |
| 194 | DTYMK |  |  | 774 | -0.522 | -0.5454 | Yes |
| 195 | RFC4 |  |  | 777 | -0.527 | -0.5457 | Yes |
| 196 | STMN1 |  |  | 778 | -0.529 | -0.5428 | Yes |
| 197 | DEPDC1B |  |  | 779 | -0.533 | -0.5398 | Yes |
| 198 | CENPA |  |  | 782 | -0.538 | -0.5400 | Yes |
| 199 | SKA3 |  |  | 783 | -0.539 | -0.5370 | Yes |
| 200 | NCAPD2 |  |  | 784 | -0.540 | -0.5340 | Yes |
| 201 | CEP152 |  |  | 785 | -0.542 | -0.5310 | Yes |
| 202 | BLM |  |  | 786 | -0.543 | -0.5279 | Yes |
| 203 | FANCD2 |  |  | 787 | -0.544 | -0.5249 | Yes |
| 204 | INCENP |  |  | 789 | -0.558 | -0.5234 | Yes |
| 205 | NCAPD3 |  |  | 790 | -0.559 | -0.5203 | Yes |
| 206 | ATAD5 |  |  | 791 | -0.559 | -0.5171 | Yes |
| 207 | HAUS8 |  |  | 792 | -0.560 | -0.5140 | Yes |
| 208 | CDC20 |  |  | 793 | -0.562 | -0.5109 | Yes |
| 209 | HMGN2 |  |  | 795 | -0.564 | -0.5093 | Yes |
| 210 | RACGAP1 |  |  | 796 | -0.566 | -0.5062 | Yes |
| 211 | HMGB1 |  |  | 797 | -0.567 | -0.5030 | Yes |
| 212 | CENPJ |  |  | 799 | -0.568 | -0.5014 | Yes |
| 213 | HES6 |  |  | 800 | -0.569 | -0.4983 | Yes |
| 214 | RFC3 |  |  | 801 | -0.569 | -0.4951 | Yes |
| 215 | TUBB |  |  | 802 | -0.570 | -0.4919 | Yes |
| 216 | CEP78 |  |  | 803 | -0.570 | -0.4887 | Yes |
| 217 | TUBB4B |  |  | 804 | -0.573 | -0.4855 | Yes |
| 218 | CENPN |  |  | 805 | -0.574 | -0.4823 | Yes |
| 219 | USP1 |  |  | 806 | -0.575 | -0.4791 | Yes |
| 220 | CDCA8 |  |  | 808 | -0.585 | -0.4774 | Yes |
| 221 | PLK1 |  |  | 809 | -0.586 | -0.4741 | Yes |
| 222 | CHAF1A |  |  | 810 | -0.588 | -0.4709 | Yes |
| 223 | DEK |  |  | 812 | -0.591 | -0.4692 | Yes |
| 224 | FOXM1 |  |  | 814 | -0.593 | -0.4675 | Yes |
| 225 | DEPDC1 |  |  | 815 | -0.596 | -0.4641 | Yes |
| 226 | CDCA2 |  |  | 816 | -0.597 | -0.4608 | Yes |
| 227 | LMNB1 |  |  | 818 | -0.606 | -0.4590 | Yes |
| 228 | MCM8 |  |  | 819 | -0.607 | -0.4556 | Yes |
| 229 | MND1 |  |  | 821 | -0.615 | -0.4538 | Yes |
| 230 | PIF1 |  |  | 822 | -0.616 | -0.4504 | Yes |
| 231 | AURKA |  |  | 824 | -0.623 | -0.4485 | Yes |
| 232 | CKAP2L |  |  | 826 | -0.625 | -0.4466 | Yes |
| 233 | CCDC34 |  |  | 827 | -0.626 | -0.4431 | Yes |
| 234 | DNAJC9 |  |  | 828 | -0.633 | -0.4396 | Yes |
| 235 | AURKB |  |  | 829 | -0.634 | -0.4361 | Yes |
| 236 | HELLS |  |  | 830 | -0.635 | -0.4325 | Yes |
| 237 | DTL |  |  | 831 | -0.637 | -0.4289 | Yes |
| 238 | PCNA |  |  | 832 | -0.640 | -0.4254 | Yes |
| 239 | BUB3 |  |  | 834 | -0.641 | -0.4234 | Yes |
| 240 | CENPW |  |  | 835 | -0.643 | -0.4198 | Yes |
| 241 | CDKN3 |  |  | 836 | -0.653 | -0.4162 | Yes |
| 242 | BUB1 |  |  | 837 | -0.653 | -0.4125 | Yes |
| 243 | ITGB3BP |  |  | 838 | -0.661 | -0.4088 | Yes |
| 244 | E2F7 |  |  | 840 | -0.675 | -0.4067 | Yes |
| 245 | ANP32E |  |  | 841 | -0.676 | -0.4029 | Yes |
| 246 | KIF22 |  |  | 842 | -0.678 | -0.3991 | Yes |
| 247 | BIRC5 |  |  | 843 | -0.679 | -0.3953 | Yes |
| 248 | CENPM |  |  | 844 | -0.682 | -0.3915 | Yes |
| 249 | NCAPH |  |  | 845 | -0.685 | -0.3877 | Yes |
| 250 | BRCA1 |  |  | 846 | -0.691 | -0.3838 | Yes |
| 251 | ZWINT |  |  | 847 | -0.692 | -0.3799 | Yes |
| 252 | SPC24 |  |  | 848 | -0.694 | -0.3761 | Yes |
| 253 | BRCA2 |  |  | 850 | -0.694 | -0.3738 | Yes |
| 254 | VRK1 |  |  | 852 | -0.711 | -0.3714 | Yes |
| 255 | ECT2 |  |  | 854 | -0.717 | -0.3690 | Yes |
| 256 | KIF2C |  |  | 855 | -0.721 | -0.3650 | Yes |
| 257 | NUF2 |  |  | 856 | -0.721 | -0.3610 | Yes |
| 258 | CENPK |  |  | 857 | -0.722 | -0.3569 | Yes |
| 259 | FAM111A |  |  | 858 | -0.727 | -0.3529 | Yes |
| 260 | SYNE2 |  |  | 859 | -0.728 | -0.3488 | Yes |
| 261 | ANLN |  |  | 860 | -0.733 | -0.3447 | Yes |
| 262 | KIF4A |  |  | 861 | -0.736 | -0.3406 | Yes |
| 263 | HMMR |  |  | 863 | -0.740 | -0.3381 | Yes |
| 264 | FBXO5 |  |  | 864 | -0.742 | -0.3339 | Yes |
| 265 | CCNB2 |  |  | 865 | -0.745 | -0.3298 | Yes |
| 266 | RRM1 |  |  | 866 | -0.749 | -0.3256 | Yes |
| 267 | ARHGAP11A |  |  | 867 | -0.756 | -0.3214 | Yes |
| 268 | NDC80 |  |  | 869 | -0.762 | -0.3187 | Yes |
| 269 | ORC6 |  |  | 870 | -0.766 | -0.3144 | Yes |
| 270 | BUB1B |  |  | 871 | -0.766 | -0.3102 | Yes |
| 271 | CCNA2 |  |  | 872 | -0.770 | -0.3058 | Yes |
| 272 | KIF23 |  |  | 873 | -0.772 | -0.3015 | Yes |
| 273 | MELK |  |  | 874 | -0.776 | -0.2972 | Yes |
| 274 | TMPO |  |  | 875 | -0.778 | -0.2928 | Yes |
| 275 | KIF20B |  |  | 876 | -0.779 | -0.2885 | Yes |
| 276 | CEP55 |  |  | 877 | -0.780 | -0.2841 | Yes |
| 277 | CKAP2 |  |  | 878 | -0.781 | -0.2798 | Yes |
| 278 | NCAPG2 |  |  | 879 | -0.781 | -0.2754 | Yes |
| 279 | PTTG1 |  |  | 880 | -0.791 | -0.2710 | Yes |
| 280 | MCM10 |  |  | 881 | -0.798 | -0.2665 | Yes |
| 281 | H2AFX |  |  | 882 | -0.799 | -0.2620 | Yes |
| 282 | KIFC1 |  |  | 883 | -0.807 | -0.2575 | Yes |
| 283 | KIF14 |  |  | 885 | -0.809 | -0.2546 | Yes |
| 284 | EZH2 |  |  | 886 | -0.810 | -0.2501 | Yes |
| 285 | KIF15 |  |  | 890 | -0.834 | -0.2503 | Yes |
| 286 | CDCA5 |  |  | 892 | -0.841 | -0.2472 | Yes |
| 287 | H2AFZ |  |  | 893 | -0.849 | -0.2425 | Yes |
| 288 | RAD51AP1 |  |  | 894 | -0.853 | -0.2377 | Yes |
| 289 | KPNA2 |  |  | 895 | -0.858 | -0.2329 | Yes |
| 290 | DBF4 |  |  | 896 | -0.864 | -0.2281 | Yes |
| 291 | SMC2 |  |  | 897 | -0.867 | -0.2232 | Yes |
| 292 | CENPE |  |  | 899 | -0.873 | -0.2200 | Yes |
| 293 | PBK |  |  | 900 | -0.880 | -0.2150 | Yes |
| 294 | FANCI |  |  | 901 | -0.882 | -0.2101 | Yes |
| 295 | SMC4 |  |  | 902 | -0.886 | -0.2052 | Yes |
| 296 | MIS18BP1 |  |  | 904 | -0.889 | -0.2018 | Yes |
| 297 | C21orf58 |  |  | 905 | -0.893 | -0.1968 | Yes |
| 298 | CCNB1 |  |  | 906 | -0.895 | -0.1918 | Yes |
| 299 | XRCC2 |  |  | 908 | -0.907 | -0.1884 | Yes |
| 300 | CKS1B |  |  | 909 | -0.912 | -0.1833 | Yes |
| 301 | HJURP |  |  | 910 | -0.921 | -0.1781 | Yes |
| 302 | CKS2 |  |  | 911 | -0.922 | -0.1730 | Yes |
| 303 | GTSE1 |  |  | 912 | -0.941 | -0.1677 | Yes |
| 304 | KIF11 |  |  | 913 | -0.957 | -0.1623 | Yes |
| 305 | CLSPN |  |  | 916 | -0.964 | -0.1602 | Yes |
| 306 | TYMS |  |  | 917 | -0.965 | -0.1548 | Yes |
| 307 | CENPU |  |  | 918 | -0.971 | -0.1494 | Yes |
| 308 | PRC1 |  |  | 919 | -0.980 | -0.1439 | Yes |
| 309 | MAD2L1 |  |  | 921 | -0.990 | -0.1400 | Yes |
| 310 | UBE2T |  |  | 922 | -0.994 | -0.1344 | Yes |
| 311 | DLGAP5 |  |  | 923 | -0.998 | -0.1288 | Yes |
| 312 | SPC25 |  |  | 924 | -1.033 | -0.1231 | Yes |
| 313 | ESCO2 |  |  | 926 | -1.067 | -0.1187 | Yes |
| 314 | UBE2C |  |  | 928 | -1.108 | -0.1141 | Yes |
| 315 | NUSAP1 |  |  | 929 | -1.112 | -0.1079 | Yes |
| 316 | TPX2 |  |  | 930 | -1.124 | -0.1016 | Yes |
| 317 | NCAPG |  |  | 931 | -1.142 | -0.0953 | Yes |
| 318 | ASPM |  |  | 932 | -1.146 | -0.0888 | Yes |
| 319 | ATAD2 |  |  | 933 | -1.162 | -0.0824 | Yes |
| 320 | CENPF |  |  | 935 | -1.200 | -0.0773 | Yes |
| 321 | HIST1H1C |  |  | 936 | -1.220 | -0.0704 | Yes |
| 322 | HMGB2 |  |  | 937 | -1.238 | -0.0635 | Yes |
| 323 | CDK1 |  |  | 938 | -1.248 | -0.0565 | Yes |
| 324 | RRM2 |  |  | 940 | -1.403 | -0.0503 | Yes |
| 325 | MKI67 |  |  | 941 | -1.407 | -0.0425 | Yes |
| 326 | TOP2A |  |  | 942 | -1.417 | -0.0345 | Yes |
| 327 | HIST1H1D |  |  | 944 | -1.810 | -0.0260 | Yes |
| 328 | HIST1H1B |  |  | 945 | -1.903 | -0.0154 | Yes |
| 329 | HIST1H4C |  |  | 946 | -2.752 | -0.0000 | Yes |
Table: GSEA details [plain text format]

  

Fig 2: RICHARDS\_NOWAKOWSKI\_SCIENCE\_2017\_IPC\_DIV1\_UPREG: Random ES distribution      
 Gene set null distribution of ES for **RICHARDS\_NOWAKOWSKI\_SCIENCE\_2017\_IPC\_DIV1\_UPREG**
